# Variations in risk assessment models may contribute to the existing gap between venous thromboembolism prophylaxis guidelines and adherence

**DOI:** 10.1186/2193-1801-1-60

**Published:** 2012-12-11

**Authors:** Deepti Vyas

**Affiliations:** Assistant Professor, Pharmacy Practice Department, Thomas J. Long School of Pharmacy and Health Sciences, University of the Pacific, 751 Brookside Road, Stockton, CA 95207 USA

**Keywords:** Deep vein thrombosis, Risk assessment model, Venous thromboembolism, Risk assessment protocol, Thromboprophylaxis

## Abstract

**Background:**

Risk assessment models (RAMs) may allow the clinician to determine need for deep vein thrombosis (DVT) prophylaxis. Individual healthcare facilities often develop their own RAMs. The purpose of this study was to determine: 1.) inter-RAM variability in DVT risk factors and contraindications; 2.) inter-rater variability and inter-RAM variability when applying a RAM to a standard case; and 3.) inter-rater and inter-RAM variability in outcome as far as type of prophylaxis. A convenience sample of RAMs was obtained from various institutions and ten reviewers were recruited to apply the RAMs to three patient cases.

**Finding:**

The review resulted in 390 separate assessments. Patient 1 did not receive any chemoprophylaxis in 67% of the evaluations, patient 2 in 27% of the evaluations and patient 3 in 2.3% of the evaluations. There was statistically significant variation in the provision of chemoprophylaxis per RAM for patient 1 (p=0.001) and no significant variation for patients 2 and 3. When analyzing the rate of chemoprophylaxis per reviewer, there was statistically significant variation for patients 1 and 2 (p=0.026 and <0.0001 respectively) but not for patient 3 (p=0.123).

**Conclusion:**

There may be significant inter-RAM and inter-reviewer variability when utilizing RAMs for assessing DVT risk.

## Background

Among hospitalized patients, venous thromboembolism (VTE) represents the second most common nosocomial condition, the second most common reason for an increased length of hospital stay, and the third most common cause of increased mortality (Zhan & Miller 2003). Given the high morbidity and mortality associated with this disease, the American College of Chest Physicians (ACCP) calls for preventive strategies and risk assessment for all admitted patients. (Kahn et al. 2012) However, despite the many published recommendations there appears to be a widening gap between guidelines and implementation of preventative strategies. The IMPROVE study showed that utilization of prophylaxis for admitted medical patients was around 60% among 15,000 hospitalized patients. (Tapson et al. 2007) The ENDORSE study found an even lower number (less than 40%) among at risk medical patients (Cohen et al. 2008). According to the 9^th^ ACCP guidelines, all medical patients on bed-rest with at least one additional risk factor should receive chemoprophylaxis while those at a high risk of bleeding should receive mechanical prophylaxis with graduated compression stockings (GCS) or intermittent pneumatic compression devices (IPCs). (Kahn et al. 2012)

There are several strategies for improving the provision of VTE prophylaxis including: 1.) Risk assessment models (RAMs); 2.) Risk recognition strategies; and 3.) Universal prophylaxis (Nutescu 2007). The 9^th^ ACCP guidelines favor risk recognition/assessment over universal prophylaxis. (Kahn et al. 2012) RAMs may allow the clinician to assess the need for thromboprophylaxis and determine appropriate prophylaxis based on individual risk factors. RAMs for identifying patients at risk for VTE have been described in the literature (Arcelus et al. 1991; Cohen & Alikhan 2001; Thromboembolic Risk Factors (THRIFT) Consensus Group 1992; Caprini et al. 2001). RAMs vary greatly from ease of use, risk factor stratification, and decision support. Some RAMs may provide the clinician with a risk score but do not provide guidance regarding which prophylaxis fits the patient’s needs. Others provide decision support and guide the clinician toward either mechanical or chemoprophylaxis. In general however, these RAMs may be time consuming, lack internal validity, be difficult to use, and lack generalizability for all patient populations (Kahn et al. 2012). However, despite this, RAMs continue to be used at healthcare institutions.

This paper presents an exploratory study looking at a convenience sample of RAMs to evaluate the reliability of these tools in assessing medical patients’ risk for DVT and clinical decision support with regard to appropriate thromboprophylaxis. This study focused on medical patients as this patient population has the greatest variability in terms of risk factors for DVT. The objectives were to determine: 1.) inter-RAM variability in DVT risk factors and contraindications; 2.) inter-rater variability and inter-RAM variability when applying a RAM to a standard case; and 3.) inter-rater and inter-RAM variability in outcome as far as type of prophylaxis.

## Methods and materials

A convenience sample of DVT RAMs was obtained from various institutions in the United States. Several e-mail requests for RAMs were sent to the American College of Clinical Pharmacy Adult medicine listserve that caters to about a thousand pharmacist subscribers from various institutions. The primary investigator reviewed the RAMs, seven of which were duplicate, incomplete or catered toward the surgical population and were subsequently discarded. Thirteen RAMs from various institutions were deemed appropriate and all institutional identifiers were removed from the RAMs to avoid bias. Four faculty members were recruited to provide an initial review of the various RAMs in determining patient eligibility for DVT prophylaxis. The 4 reviewers conducted an exploratory review of the thirteen RAMs to determine any variations in risk factors for DVT and contraindications to chemoprophylaxis within the RAMs. Based on this preliminary review, it was determined that significant variation existed between various RAMs which warranted a further review.

### Phase 2 RAM evaluation

Ten healthcare professionals, 5 physicians and 5 pharmacists, were recruited. Three patient cases were developed and only the admission data was provided to the reviewers, as most decisions regarding DVT prophylaxis are made at admission (Table 1). The clinicians were given the 13 RAMs and were asked to apply each RAM to the three patient cases to determine each patient’s risk for developing a DVT while in the hospital. Based on the patient’s risk assessment, the clinicians were then asked to determine the most appropriate DVT prophylaxis as directed by the RAM. They worked independently to avoid bias. Differences in risk assessment scoring per RAM, scoring per reviewer and treatment choices were then analyzed.

**Table 1:** **Standard patient cases**

	Patient case 1	Patient case 2	Patient case 3
**CC**	Two week history of constipation.	Nausea, vomiting, and high blood pressure.	Pneumonia.
	*Admitted to General medicine service.*	*Admitted to General medicine service*	*Admitted to General medicine service.*
**HPI**	18 year old female with chronic history of pelvic floor dysfunction, who presents with chronic constipation for 14 days, associated with nausea, vomiting, and abdominal pain.	19 year old female who is transferred from an outside hospital with nausea, vomiting, and unable to control blood sugars	58 year old male who was admitted after complaining of "shortness of breath." Patient was found to have pneumonia and pulmonic valve agitation consistent with endocarditis
**PMH**	Asthma	Type 1 Diabetes Mellitus	ESRD status-post cadaveric renal transplant
	Depression	SLE	Hypertension.
	Fibromyalgia	JRA	Non-insulin-dependent diabetes mellitus
	Chronic constipation	Asthma	Dyslipidemia.
	TMJ disorder	Raynaud's	Renal transplant.
	Pelvic pain	Duodenitis	Multiple AV grafts.
		Gastroparesis	
		Celiac sprue	
		Bursitis of both hips	
**FH**	NR	NR	NR
**SH**	NR	NR	NR
**Home medications**	Prozac 80 mg po daily.	Venlafaxine 150 mg po qAM	Atenolol 100 PO daily
	Ortho-tri-cylen 1 po daily	Nifedipine 30 mg po qAM	Lipitor 10 mg PO daily
	Magnesium chelate 40 mg po daily	Lisinopril 5 mg po Daily	Cyclosporine 75 mg PO bid
	Lactobacillus 1 capsule po daily	Hydroxychloroquine 200 mg po bid	Diltiazem 300 mg PO daily
	Lyrica 150 mg po bid	Fluticasone 1 Puff Daily	Lasix 40 mg PO daily
	Abilify 6 mg po daily	Esomeprazole 40 mg po bid	Glipizide 5 mg PO bid
	Pimozide 1 mg po daily	Clonazepam 2 mg po daily	Lisinopril 15 mg PO bid
	Tizanidine 4–8 mg po bid prn	Albuterol 4 Puffs q1hour PRN	Losartan 100 mg PO daily
		Insulin pump	Cellcept 500 mg PO bid
			K-Phos 250 mg PO bid
			Prednisone 10 mg every other day PO
**Laboratory data**	WBC: 8.1, Hgb: 12.6, Plt: 277, Na 137, K:3.6, Cl:104, Hco3:24, anion gap 13, Gluc: 90, BUN:10, Cr: 0.73, Ca: 9.1, protein 7.8, Alb: 4.3, total bilirubin: 0.5, Alk phos: 50, AST 21, ALT 13	Not available on admission	Not available on admission
**Height and weight**	Height: 175 cm	Height: 154.9 cm	Height: Not available
	Weight: 69 kg	Weight:74 kg	Weight: 135 kg

*CC* Chief complaint, *HPI* History of present illness, *PMH* Past medical history, *TMJ* Temporomandibular joint, *SLE* Systemic lupus erythematosus, *JRA* Juvenile rheumatoid arthritis, *ESRD* End stage renal disease, *AV* Arteriovenous, *NR*=Non-remarkable, *Po* Oral. *mg* milligrams, *BID* Twice daily, *PRN* As needed, *qAM* Every morning, *WBC* White blood cell count, *Hgb* Hemoglobin, *Plt* Platelets, *Na* Sodium, *K* Potassium, *Cl* Chloride, *HCO3* Bicarbonate, *Gluc* Glucose, *BUN* Blood Urea Nitrogen, *Cr* Creatinine, *Ca* Calcium, *Alb* Albumin, *Alk phos* alkaline phosphatase, *AST* Aspartate aminotransferase, *ALT* Alanine transaminase, *cm* Centimetres, *kg* Kilogram.

### Statistical analysis

Descriptive statistics were used to analyze some data. Cohen’s kappa was used to assess the pairwise agreement for each of the 45 possible pairs of reviewers. The distribution of these values was graphically explored using a histogram. Following this, a multidimensional plot of the kappa values was constructed to provide a visual representation of proximity among the reviewers. A mixed model logistic regression analysis was used to test for a statistically significant difference among the various RAMs. The patients and RAM was analyzed as a fixed variable. Finally, an inter-rater reliability score for the ten reviewers was further assessed in a two-way analysis of variance (ANOVA) model with the factors being patient and reviewer.

## Results

Preliminary review of the RAMs showed differences which precipitated further analysis of the RAMs by various clinicians. The type and number of risk factors varied greatly within each RAM. RAMs also differed in the number and type of contraindications to chemoprophylaxis. In total, there were 66 different risk factors (range 6–31) identified in the RAMs and there were 39 different contraindications to chemoprophylaxis (range 1–17). There were significant variations in the definitions of certain risk factors especially with regard to age and obesity. Some RAMs defined elderly as age >60 (61%) whereas other RAMs defined it as age >75 (15%). Some RAMs defined obesity as a body mass index (BMI) of > 30 (31%), while some defined it as a BMI>50 (8%).

**Figure 1:**
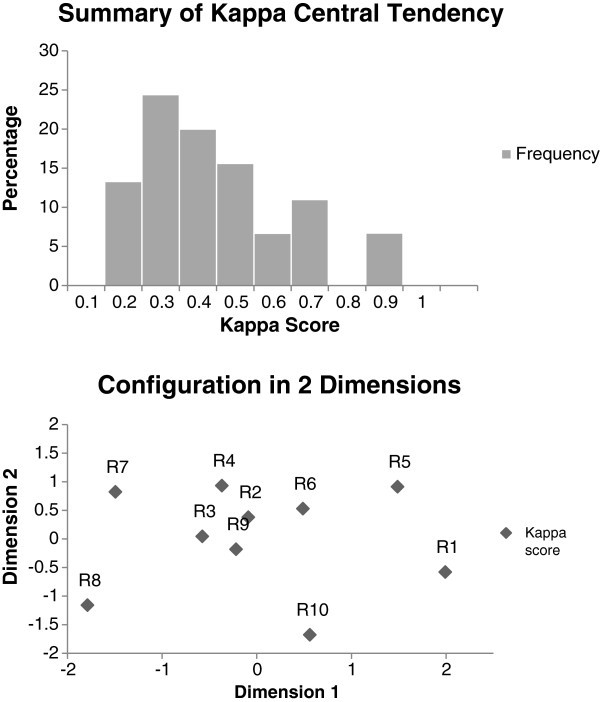
**Multidimensional plot displaying the spread of reviewer agreement and pairwise inter-rater agreement when applying various RAMs to a patient case.**

The phase 2 review resulted in 390 separate assessments. Use of the RAMs on the 3 patient cases revealed significant inter-rater and inter-RAM variability. Kappa scores are displayed in Figure 1 which showed significant disagreement between reviewers when applying each RAM to a standard patient case with a central tendency of 0.2-0.4. A multidimensional plot was created which displayed the significant spread of reviewer agreement (Figure 1). There was statistically significant difference in the RAM effect when applied to each patient case (p=0.01). Patient 1 did not receive any chemoprophylaxis in 67% of the evaluations (range 0-80%), patient 2 in 27% of the evaluations (range 10-50%) and patient 3 in 2.3% of the evaluations (range 0-10%). There was statistically significant variation in the provision of chemoprophylaxis per RAM for patient 1 (p=0.001) and no significant variation for patients 2 and 3. When analyzing the rate of chemoprophylaxis per reviewer, there was statistically significant variation for patients 1 and 2 (p=0.026 and <0.0001 respectively) but not for patient 3 (p=0.123). On average, each reviewer spent 5–10 minutes to review the RAM and determine the most appropriate prophylaxis based on the patient characteristics.

## Discussion

This pilot study found significant inter-rater and inter-RAM variability with use of RAMs from various US healthcare institutions. Based on this initial data, it is clear there is a probability that there will be considerable variation in the ordering practices of DVT prophylaxis with the use of traditional RAMs. It is also clear that there is variation in the type and number of risk factors associated with DVTs. The new ACCP guidelines now elucidate that the risk factors for VTE in medical patients include increasing age (especially > 70 years), previous VTE, known thrombophilia, active cancer, heart failure, or respiratory failure, reduced mobility, and hormonal medications. The guidelines also provide a scoring system for these risk factors which should theoretically result in consistent risk assessment for all patients. In order to maintain consistency, using the risk factor and scoring elucidated by the guidelines may reduce the variability in the provision of DVT prophylaxis however there may still be possibility of inter-rater variability especially in terms of ambiguous risk factors such as ‘reduced mobility, recent trauma/surgery’ as well as ‘heart and respiratory failure’. In addition, the ACCP guidelines, state that those patients who are deemed high risk for bleeding should receive mechanical prophylaxis. High risk patient are those with multiple risk factors for bleeding or those with an active gastroduodenal ulcer, bleeding in the 3 months before admission, or a platelet count <50 × 10^9^/L. These contraindications for chemoprophylaxis leave room for provider interpretation especially with regard to ambiguous factors such as ‘bleeding in 3 months before admission’ or even interpretation of ‘multiple’ risk factors. As a case in point, would an 85 year old male who is immobile with a history of heart failure be considered high risk for bleeding? In this paper, we showed that there was considerable inter-rater variability even when the same RAM was utilized, this means that even though the guidelines have now clearly identified a risk scoring system, there still may be potential of missed prophylaxis for patients that would benefit from receiving prophylaxis. Individual institutions will have to identify the best way to determine risk for DVT. In this paper, we argue that RAMs may not be the best approach to identifying these patients.

### Study strengths and limitations

This was a small exploratory study that highlights the limitations of traditional RAMs. This study has provided some preliminary data indicated significant inter-rater and inter-RAM variability and more importantly resulted in suboptimal DVT prophylaxis rates. However, more rigorous studies would have to be performed to establish or refute the routine use of RAMs in clinical practice. In addition, this study was conducted prior to the release of the 9^th^ ACCP guidelines and it is possible that institutions have since updated their RAMs to include only those risk factors identified by ACCP as being significant for the development of a DVT.

The number of reviewers and types of reviewers was also small, which is a significant limitation of this study. The lack of inclusion of reviewers from the nursing profession is a limitation because sometimes nursing staff are responsible for performing risk assessment for DVT prophylaxis. More reviewers and RAMs would have improved the strength of the study.

## Conclusion

This exploratory study has shown that RAMs may not be the ideal tool for determining the appropriate DVT prophylaxis for hospitalized patients and may result in significant inter-rater variability and suboptimal provision of DVT prophylaxis. Variation in RAMs may result in missed opportunities for providing appropriate prophylaxis to medical patients.
